# Dietary Multivitamin Supplementation in *Siganus fuscescens*: Integrated Effects on Growth Performance, Antioxidant Capacity, and Mitigation of Darkening Syndrome Under High-Lipid Dietary Stress

**DOI:** 10.3390/ani16213495

**Published:** 2026-11-05

**Authors:** Xin Li, Zhengbang Chen, Xuexi Wang, Xinhua Chen, Lianghua He, Yinnan Mu, Pan Qin

**Affiliations:** 1 State Key Laboratory of Mariculture Breeding, Key Laboratory of Marine Biotechnology of Fujian Province, College of Marine Sciences, Fujian Agriculture and Forestry University, Fuzhou 350002, China; 2 Fuzhou Haima Feed Co., Ltd., Fuzhou 350311, China

**Keywords:** *Siganus fuscescens*, lipid level, darkening syndrome, serum parameters, antioxidant capacity

## Abstract

*Siganus fuscescens*, an economically important species in southern China, is noted for its rapid growth and high nutritional value. Nonetheless, the prevalence of darkening syndrome, a condition marked by skin discoloration and significant mortality rates, presents a challenge to aquaculture practices, with its etiology remaining largely unexplored. This study aimed to examine the impacts of varying dietary lipid levels and multivitamin supplementation on the incidence of darkening syndrome in *S. fuscescens*. Five experimental diets were developed: a low-fat diet containing 10% lipids; a moderate-fat diet with 13% lipids; a moderate-fat diet with 13% lipids supplemented with multivitamins; a high-fat diet containing 16% lipids; and a high-fat diet with 16% lipids supplemented with multivitamins. A 55-day feeding trial was conducted on *S. fuscescens* specimens. The results indicate that, in this experiment, increasing dietary fat content led to a higher cumulative incidence of darkening syndrome in *S. fuscescens*. Serological and histological findings suggested that in the 16% lipid diet group, *S. fuscescens* exhibited liver injury and oxidative stress. Notably, supplementation with multivitamins alleviated these adverse effects. Overall, we recommend using an appropriate dietary fat level and further supplementing with antioxidant multivitamins, which may represent an effective strategy to mitigate darkening syndrome in *S. fuscescens*.

## Simple Summary

Darkening syndrome is a major problem in farming *Siganus fuscescens* in southern China, causing skin to turn darker and leading to many deaths, but its causes are not fully understood. We tested whether the amount of fat in fish feed and the addition of multivitamins could change the risk of the disease. Over a 55-day feeding trial, fish were given one of five diets: low fat (10%), moderate fat (13%), moderate fat (13%) plus multivitamins, high fat (16%), or high fat (16%) plus multivitamins. In this study, we found that the incidence of darkening syndrome increased with increasing dietary fat levels in *S. fuscescens*. However, these negative effects were alleviated by the supplementation of multivitamins. Therefore, an appropriate dietary fat level, together with antioxidant multivitamins, may be an effective strategy to mitigate darkening syndrome in *S. fuscescens*.

## 1. Introduction

Darkening syndrome is an idiopathic pathological condition affecting commercially cultured marine teleosts, characterized by abnormal skin darkening, reduced activity, and loss of equilibrium. In addition to these factors, the syndrome involves physiological processes related to skin pigmentation. In teleosts, skin darkening is primarily influenced by compositional and structural changes in dermal melanophores [1], which produce dark or brown melanin granules and establish patterns of melanin deposition [1]. Melanin also plays a role in scavenging and quenching reactive oxygen species (ROS) [2], suggesting a potential link between pigmentation and oxidative stress status. Darkening syndrome often leads to mass mortality events, resulting in significant economic losses. Its widespread occurrence in commercial cultivation has become a critical bottleneck to the sustainable development of the industry. Despite its severe impact, the etiology of darkening syndrome in marine teleosts remains poorly understood. Despite the limited number of dedicated studies on darkening syndrome in marine teleosts, existing research on melanosis suggests that pathogen infections and dietary composition are prevalent contributing this condition across various fish species. Zhu et al. [3] documented that juvenile Asian seabass (*Lates calcarifer*) infected with megalocytivirus exhibited symptoms such as darkened body coloration, lethargy, and frequent surfacing or circling along pond edges during the epidemic. Similarly, silver pomfret [*Pampus argenteus* (Euphrasén, 1788)] infected with iridovirus displayed darkened body coloration and swimming imbalance [4]. Furthermore, Zhang et al. [5] demonstrated that the skin hue of channel catfish (*Ictalurus punctatus*) was affected by the consumption of oxidized fish oil, although this effect was partially alleviated by chlorogenic acid supplementation. Collectively, these observations indicate that infectious agents and nutritional composition are prevalent contributors to the etiology of darkening syndrome.

*Siganus fuscescens* is a prevalent marine herbivorous fish [6] with an optimal growth temperature range of 20–30 °C. It holds significant commercial value in southern China, due to its rapid growth, excellent palatability, and high levels of polyunsaturated fatty acids [7,8,9]. Despite its economic importance, the occurrence of darkening syndrome poses a common challenge in its cultivation, particularly when summer water temperatures are elevated in July–August each year. But research on this issue remains sparse. In response to escalating feed costs, the aquaculture industry frequently increases dietary lipid levels in species such as *S. fuscescens* [10]. However, this cost-reduction strategy may inadvertently increase oxidative stress and exacerbate the incidence of darkening syndrome [11,12].

Based on these associations, dietary antioxidant supplementation emerges as a logical intervention. Vitamins A, C, and E form a synergistic antioxidant network [13,14,15] and have been shown to reduce oxidative stress, enhance immune function, and improve growth performance in various farmed fish species [16,17,18]. Additionally, vitamin E enhances immunity in ♀*E. fuscoguttatus* × ♂*E. lanceolatus* under high-fat feeding conditions by reducing oxidative damage, upregulating anti-inflammatory factors, and downregulating pro-inflammatory factors [19]. Furthermore, research has shown that vitamin E, especially when combined with vitamin C, enhances growth and survival in species such as *Pagrus major* [20] and the whiteleg shrimp (*Litopenaeus vannamei*) [21]. Nonetheless, the protective effectiveness of multivitamin supplementation in preventing darkening syndrome in *S. fuscescens* under high-fat dietary conditions is not yet well understood.

In this study, we conducted a comparative analysis of serum parameters between healthy *S. fuscescens* and those exhibiting darkening syndrome. We evaluated the effects of varying dietary lipid levels on growth performance, the incidence of darkening syndrome, serum biochemical parameters, and histological changes in the liver and intestines of *S. fuscescens*. Furthermore, we assessed the protective effects of multivitamin supplementation against lipid-induced oxidative stress. The findings provide a key dietary-based reference for alleviating darkening syndrome in *S. fuscescens*.

## 2. Materials and Methods

### 2.1. Experimental Diets and Management

**Figure 1:**
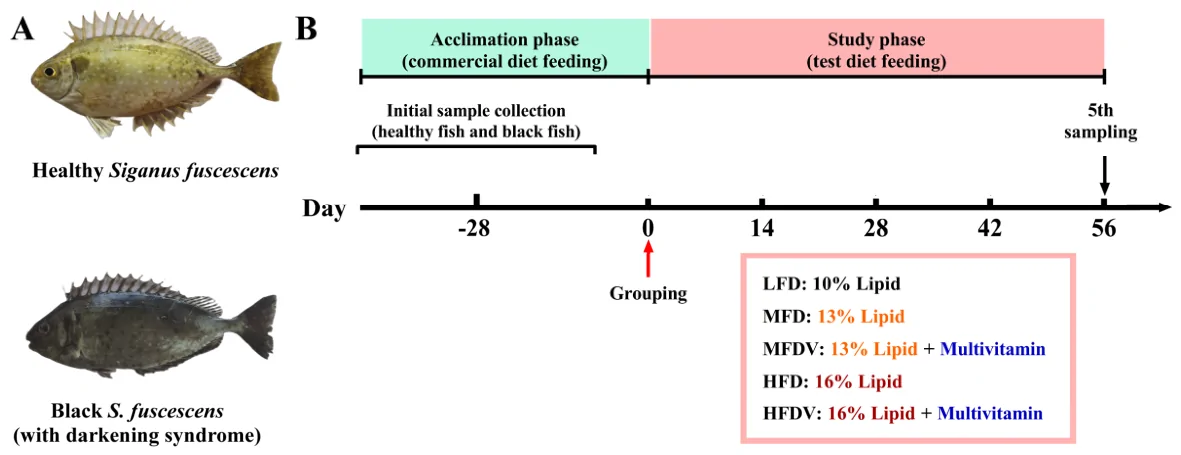
(**A**) Representative images of normal *S. fuscescens* and individuals exhibiting darkening syndrome; (**B**) experimental timeline and grouping design for the feeding trial.

The components utilized in the formulation of the experimental diets are presented in Table S1. The protein sources incorporated into these diets were fermented soybean meal, soy protein concentrate, and fish meal, while the lipid sources included soybean oil, fish oil, and phospholipids. The diets were formulated with lipid contents of 10%, 13%, and 16%, and were categorized as the low-fat diet (LFD), moderate-fat diet (MFD), and high-fat diet (HFD), respectively (Figure 1). Subgroups of the MFD and HFD were further supplemented with a multivitamin complex, resulting in the MFD with multivitamin supplementation (MFDV) and HFD with multivitamin supplementation (HFDV) to assess the protective effects of multivitamin supplementation under conditions of high lipid stress.

Solid feed components were ground using a 60-mesh screen, accurately weighed, and thoroughly mixed according to the specified formulation. The lipid blend, consisting of soybean oil, phospholipids, and fish oil, was integrated into the solid ingredients along with 30% water (*w*/*w*) and mixed until a homogeneous mixture was achieved. The resulting mixture was then extruded into pellets (Φ 4 mm), oven-dried for 1 h, and subsequently air-dried before being packaged in sealed bags and stored at −20 °C.

To minimize the influence of large-scale mortality caused by the active period of summer darkening syndrome and other periods of high pathogen activity, this study was conducted in autumn. Throughout the trial, water temperature was maintained between 15.4 and 21.1 °C, and dissolved oxygen levels were consistently above 6.0 mg/L. Experimental *S. fuscescens* specimens were acclimated in four cubic floating cages, each measuring 3.0 × 3.0 × 3.0 m, for a period of 28 days prior to commencement of the trial in Ningde, Fujian Province. During the acclimation period, the fish were fed a commercial diet. Post-acclimation, a cohort of 20,000 fish, exhibiting uniform size (mean weight: 69.69 ± 0.70 g) and free from darkening syndrome, was selected and randomly distributed into 15 floating cages, with three replicates per experimental group and 1000 fish per cage. The feeding trial, spanning eight weeks, involved twice-daily feeding at 08:00 and 14:00, continuing until the fish reached satiation, as indicated by the cessation of active feeding. The cessation of active feeding was defined as the absence of all of the following after feeding: swimming toward the feeding point, chasing feed, and ingestion of feed pellets. Feed rations for each experimental group were adjusted biweekly, informed by sampled body weights. Each biweekly sampling consisted of 30 fish.

### 2.2. Sampling

The sampling procedures are depicted in Figure 1. Prior to grouping, 10 healthy *S. fuscescens* individuals and 10 individuals exhibiting darkening syndrome were anesthetized using MS-222 to facilitate the collection of blood from the caudal vein and the dissection necessary to obtain serum and intestinal tissues. Throughout the 56-day feeding trial, daily records were meticulously maintained, documenting feed input, the number of fish displaying darkening syndrome, and mortality rates. Additionally, water temperature and dissolved oxygen levels were systematically monitored.

At the end of the 56-day feeding trial, fish were fasted for 24 h. Thirty fish per cage were randomly selected, anesthetized with MS-222, and measured for body length and body weight. After dissection, the liver, intestine (with contents removed), intraperitoneal adipose tissue, and other visceral organs were weighed. Liver and intestinal tissues were preserved in 4% paraformaldehyde for histological analysis. Blood samples were collected from the caudal vein using a 1 mL syringe, stored at 4 °C for 24 h, centrifuged at 2500 rpm for 10 min, and the serum supernatant was collected for subsequent analysis.

Daily mortality records and cases of darkening syndrome were utilized to calculate survival rates and incidence. Data on feed input and body weight were employed to determine the weight gain rate (WGR), specific growth rate (SGR), and feed efficiency rate (FER). Morphological indices, including the hepatosomatic index (HSI), viscerosomatic index (VSI), condition factor (CF), intraperitoneal fat index (IPFI), and intestosomatic index (ISI), were calculated using measurements of body length, liver weight, intestinal weight, and mesenteric fat weight. At the end of the feeding trial, *S. fuscescens* specimens exhibiting darkening syndrome were also sampled, with morphometric measurements as well as blood and tissue collection, conducted according to a consistent protocol, ensuring uniformity in subsequent processing.

### 2.3. Analysis of Serum Biochemical Parameters

A comprehensive analysis of serum biochemical parameters was conducted, encompassing the measurement of alanine aminotransferase (ALT) activity, aspartate aminotransferase (AST) activity, alkaline phosphatase (ALP) activity, complement 3 (C3) concentration, complement 4 (C4) concentration, immunoglobulin M (IgM) concentration, catalase (CAT) activity, total superoxide dismutase (T-SOD) activity, total antioxidant capacity (T-AOC) activity, glutathione peroxidase (GSH-Px) activity, malondialdehyde (MDA) concentration, oxidized glutathione (GSSG) concentration, reduced glutathione (GSH) concentration, myeloperoxidase (MPO) activity, and lysozyme (LZM) activity. These parameters were assessed utilizing commercially available kits from Nanjing Jiancheng Bioengineering Institute, Nanjing, China.

### 2.4. Histological Evaluation of Liver and Intestinal Tissues

Preserved liver and intestinal tissue specimens were subjected to dehydration, paraffin embedding, and sectioning at a thickness of 4 μm utilizing a microtome (HistoCore AUTOCUT, Leica, Wetzlar, Germany). For histological analysis, two distinct sets of liver sections were prepared: one set underwent hematoxylin–eosin (H&E) staining, while the other was stained with Oil Red O (ORO) staining. Intestinal sections were exclusively stained with H&E. The stained sections were visualized using a light microscope (ECLIPSE Ci, Nikon, Tokyo, Japan). Quantification of lipid droplets in ORO-stained sections and vacuoles in H&E-stained sections was performed by analyzing five non-overlapping fields per section using ImageJ 1.54p software. Additionally, morphometric parameters of the intestinal H&E-stained sections, such as villus length, villus width, villus area, crypt depth, villus length-to-crypt depth ratio, circular muscle thickness, longitudinal muscle thickness, and goblet cell counts, were assessed using ImageJ software.

### 2.5. Statistical Analysis

The following formulas were employed to calculate the corresponding physiological and growth indices:

WGR (%) = [(W_t_ − W_0_)/W_0_] × 100

SGR (%/day) = [(lnW_t_ − lnW_0_)/t] × 100

FER (g/g) = (W_t_ − W_0_)/F

Food intake (g/day) = F/t

where W_0_ = initial body weight (g), W_t_ = final body weight (g), t = culture days (d), and F = feed intake per fish (g).

HSI (%) = (Wₕ/W_b_) × 100

VSI (%) = (W_v_/W_b_) × 100

CF (g/cm^3^) = W_b_/L^3^

IPFI (%) = (W_f_/W_b_) × 100

ISI (%) = (W_i_/W_b_) × 100

where W_h_ = liver weight (g), W_v_ = viscera weight (g), W_b_ = body weight (g), W_f_ = intraperitoneal fat weight (g), W_i_ = intestine weight (g), and L = body length (cm).

Relative area of hepatic vacuolization or ballooning degeneration (%) = [1 − (Pixel area of non-vacuolated regions/Total pixel area of the field)] × 100

Relative area of hepatic ORO-positive (%) = (Pixel area of ORO-positive regions/Total pixel area of the field) × 100

Statistical analyses were performed using SPSS 22.0 and R 4.5.2. Data are expressed as mean ± standard error of the mean (SEM). Survival rate and darkening syndrome incidence were analyzed using the Kaplan–Meier method. Subsequently, multiple comparisons were performed using the Benjamini–Hochberg (BH) procedure. Homogeneity of variance and normality were verified prior to analysis. Differences between groups were evaluated using Student’s *t*-test, one-way analysis of variance, and Tukey’s multiple range test. A significance threshold of *p* < 0.05 was applied for statistical analysis. Different lowercase letters indicate significant differences (* *p* <  0.05 and ** *p* <  0.01).

## 3. Results

### 3.1. Biochemical Parameters of Healthy and Darkened Fish Serum

**Figure 2:**
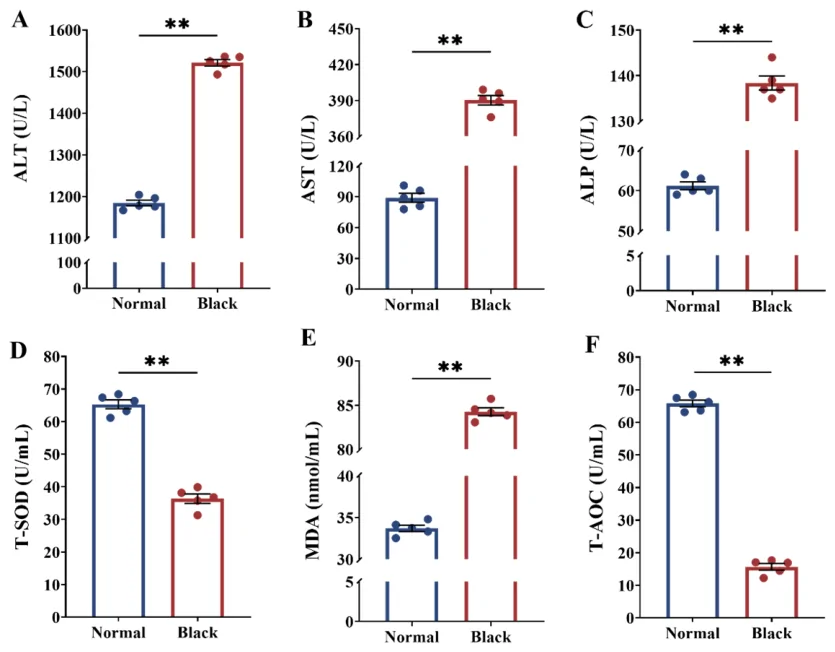
Serum biochemical parameters of normal *S. fuscescens* and those affected by darkening syndrome; (**A**) ALT, alanine aminotransferase activity; (**B**) AST, aspartate aminotransferase activity; (**C**) ALP, alkaline phosphatase activity; (**D**) T-SOD, total superoxide dismutase activity; (**E**) MDA, malondialdehyde concentration; (**F**) T-AOC, total antioxidant capacity activity; Data are expressed as mean ± SEM (*n* = 5); statistical analysis was performed using Student’s *t*-test, with significance level set at α = 0.05; ** *p*  <  0.01.

Darkening syndrome significantly affected the serum biochemical parameters of *S. fuscescens* (Figure 2 and Figure S1). Specially, serum levels of ALT, AST, ALP, and MDA were significantly elevated (*p* < 0.05) in the group afflicted with darkening syndrome compared to the healthy group. Conversely, the activities of T-SOD and T-AOC were significantly higher in the healthy group (*p* < 0.05).

The serum levels of GSSG and IgM were significantly higher (*p* < 0.05) in the darkening syndrome group compared with the healthy group. In contrast, MPO and LZM activities were significantly higher in the healthy group than in the darkening syndrome group (*p* < 0.05). Darkening syndrome exerted no significant effects on CAT, GSH-Px, GSH, and C3 levels in *S. fuscescens* (*p* > 0.05).

### 3.2. Intestinal Histological Parameters of Healthy and Darkened Fish

**Figure 3:**
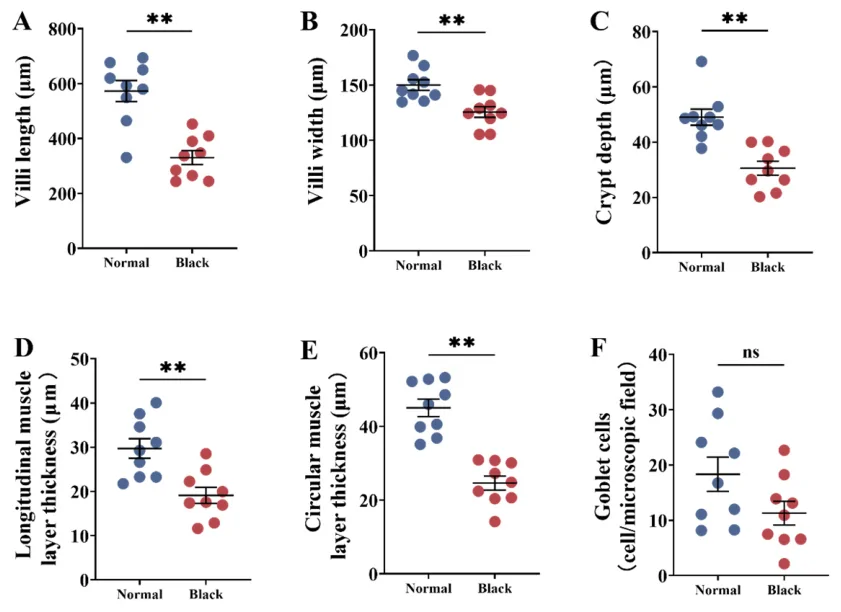
Intestinal histology and morphological parameters of normal *S. fuscescens* and those affected by darkening syndrome;(**A**) villus length; (**B**) villus width; (**C**) crypt depth; (**D**) thickness of longitudinal muscle layer; (**E**) thickness of circular muscle layer; (**F**) number goblet cells in intestinal villus; data are expressed as mean ± SEM (*n* = 9); statistical analysis was performed using Student’s *t*-test, with significance level set at α = 0.05; ns, not significant; ** *p*  <  0.01.

As illustrated in Figure 3, the darkening syndrome group exhibited significant reductions in intestinal villus length, villus width, crypt depth, and the thickness of both longitudinal and circular muscle compared with the healthy group (*p* < 0.05). No significant difference in intestinal goblet cell count was found between *S. fuscescens* affected by darkening syndrome and healthy fish (*p* > 0.05).

### 3.3. Survival Rate and Incidence of Darkening Syndrome

**Figure 4:**
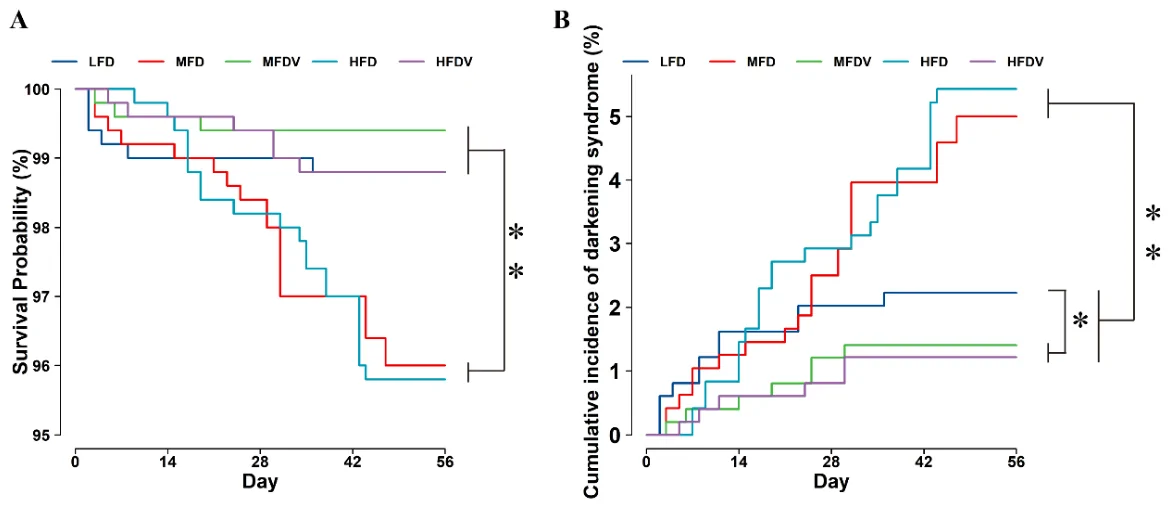
Survival rate and incidence of darkening syndrome in *S. fuscescens* fed diets with different lipid levels and multivitamin supplementation during the 56-day feeding trial. (**A**) Survival rate. (**B**) Incidence of darkening syndrome. LFD, low-fat diet; MFD, moderate-fat diet; HFD, high-fat diet; MFDV, MFD supplemented with multivitamins; HFDV, HFD supplemented with multivitamins. Survival and darkening syndrome incidence were analyzed using the Kaplan–Meier method, followed by multiple comparisons using the Benjamini–Hochberg procedure. * *p* <  0.05; ** *p*  <  0.01.

Figure 4A,B illustrate that as dietary lipid levels increased, the survival rate of *S. fuscescens* progressively decreased, while the incidence of darkening syndrome correspondingly increased. Both darkening syndrome and mortality were observed in each experimental group. The survival rates were significantly higher in the low-fat diet (LFD), moderate-fat diet with vitamin supplementation (MFDV), and high-fat diet with vitamin supplementation (HFDV) groups compared to the moderate-fat diet (MFD) and high-fat diet (HFD) groups (*p* < 0.05). Throughout the culture period, the incidence of darkening syndrome rose across all groups. However, the LFD, MFDV, and HFDV groups exhibited significantly lower incidences than the MFD and HFD groups (*p* < 0.05).

### 3.4. Growth Performance, Feed Utilization, and Histological Evaluation

Table 1 depicts the impact of varying dietary lipid levels on the growth performance and feed utilization of *S. fuscescens*. Among the groups without multivitamin supplementation, the HFD group demonstrated the highest weight gain rate (WGR), surpassing the medium-fat diet (MFD) and low-fat diet (LFD) groups. Furthermore, the HFD group exhibited superior WGR, specific growth rate (SGR), and feed efficiency ratio (FER) compared to the LFD and MFD groups, suggesting enhanced growth performance with increased dietary lipid levels. No statistically significant differences in growth performance were found between the MFD with vitamins (MFDV) and MFD groups (*p* > 0.05), nor between the HFD with vitamins and HFD groups supplemented with multivitamins. Notably, at a 13% lipid level, the MFDV group exhibited a significantly lower feed intake rate than the MFD group (*p* < 0.05). Conversely, at a 16% dietary lipid level, the HFDV group demonstrated lower final weight (FW), WGR, and SGR compared to the HFD group, although the difference in FER was not statistically significant (*p* > 0.05). These findings indicate that multivitamin supplementation did not enhance growth performance at this lipid level.

In the absence of multivitamin supplementation, the high-fat diet (HFD) group demonstrated the highest liver weight, followed by the medium-fat diet (MFD) and low-fat diet (LFD) groups. Although the differences in liver weight among these three groups were not statistically significant (*p* > 0.05), there was a discernible trend of increasing liver weight with higher lipid levels. With the introduction of multivitamin supplementation, variations in liver weight among the groups remained statistically non-significant (*p* > 0.05). At a dietary lipid level of 13%, there was no significant difference in liver weight between the MFD with vitamins (MFDV) and MFD groups (*p* > 0.05). At a 16% lipid level, the HFD with vitamins (HFDV) group showed a decreasing trend in liver weight compared to the HFD group.

### 3.5. Liver Histological Evaluation

**Figure 5:**
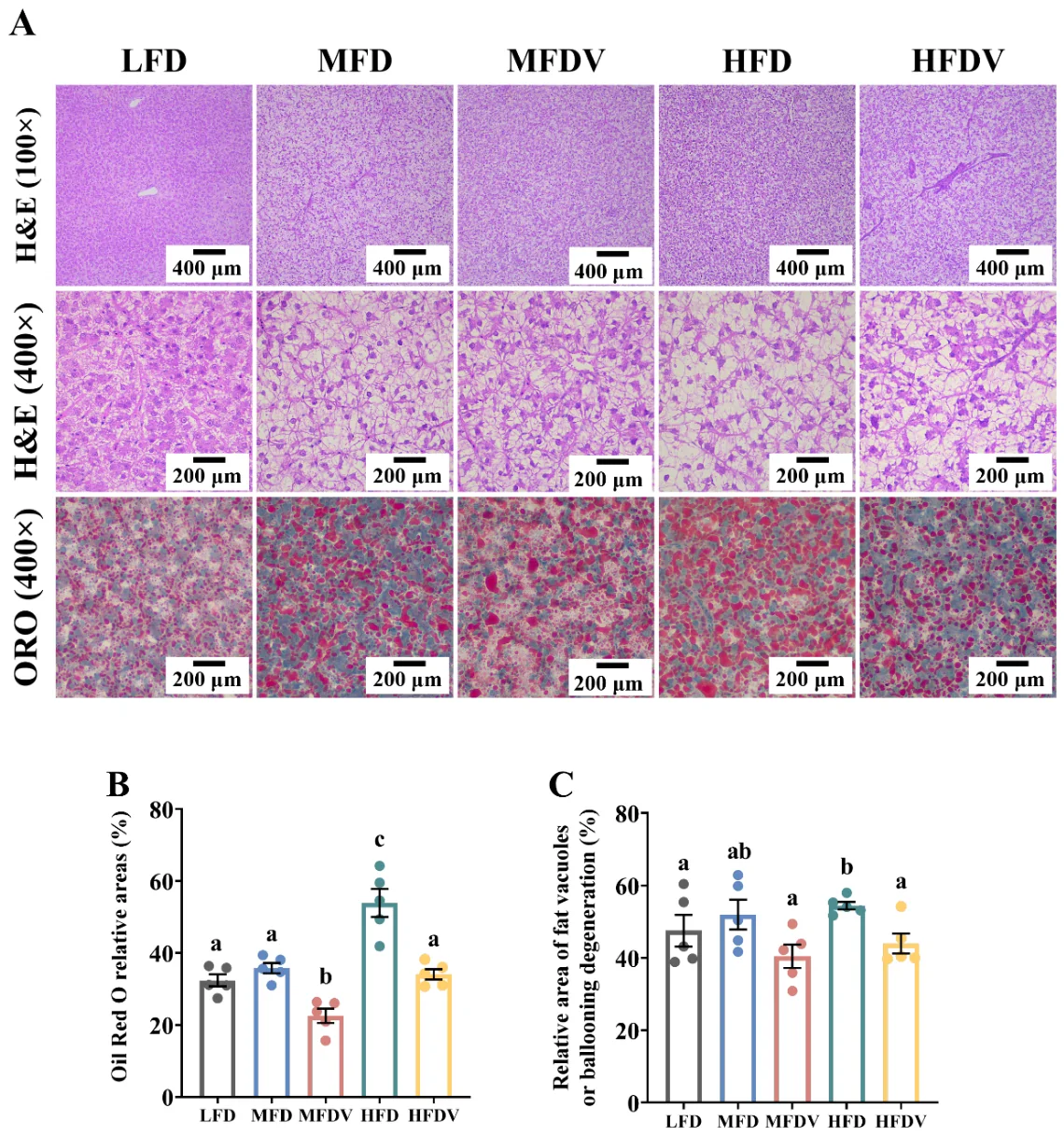
Hepatic histology of *S. fuscescens* subjected to different dietary lipid levels: (**A**) Hematoxylin-eosin (H&E) staining and Oil Red O (ORO) staining results of liver sections; (**B**) Hepatic vacuolation in different groups; (**C**) Hepatic ORO-positive areas in different groups. Statistical analysis was performed using One-way ANOVA followed by Tukey’s multiple range test, with significance level set at α = 0.05. Data are expressed as mean ± SEM (*n* = 5 per group); Values sharing identical lowercase letters indicate statistically non-significant differences (*p* > 0.05), whereas different letters represent significant differences (*p* < 0.05).

In Figure 5A, H&E staining demonstrated that increased dietary lipid levels were correlated with enhanced hepatic vacuolization. The relative area of vacuolization showed a proportional increase. Specifically, the medium-fat diet (MFD) and high-fat diet (HFD) groups exhibited hepatic relative areas of vacuolization that were 11.50% and 18.45% higher, respectively, compared to the low-fat diet (LFD) group (*p* < 0.05). Conversely, the MFD supplemented with multivitamins (MFDV) group showed a 20.07% reduction (*p* < 0.05) in hepatic relative area of vacuolization compared to the MFD group, whereas the HFD supplemented with multivitamins (HFDV) group exhibited a 17.68% decrease, although this was not statistically significant (*p* > 0.05) relative to the HFD group.

ORO staining indicated that the livers of *S. fuscescens* exhibited a progressive increase in the relative areas of lipid droplets with increasing dietary lipid levels. The hepatic relative areas of lipid droplets were 36.90% lower in the MFDV group compared to the MFD group (*p* < 0.05) and 36.82% lower in the HFDV group compared to the HFD group (*p* < 0.05).

### 3.6. Serum Hepatic Enzyme Activity

The effects of dietary lipid levels on the serum biochemical parameters of *S. fuscescens* are illustrated in Table 2. In comparison to the low-fat diet (LFD) group, the medium-fat diet (MFD) and high-fat diet (HFD) groups demonstrated 11.20% and 14.28% increases in serum alanine aminotransferase (ALT) activity, respectively (*p* < 0.05). Notably, the MFD supplemented with vitamins (MFDV) group exhibited a 47.87% reduction in serum ALT activity compared to the MFD group (*p* < 0.05), while the HFD supplemented with vitamins (HFDV) group showed a 38.81% decrease relative to the HFD group (*p* < 0.05). Serum aspartate aminotransferase (AST) activity was highest in the HFD group, showing a significant elevation compared to all other groups (*p* < 0.05). Specifically, the HFD group displayed 64.3% and 2.26-fold higher serum AST activity compared to the MFD and LFD groups, respectively (*p* < 0.05). Reductions in serum AST activity were observed in the MFDV and HFDV groups, with decreases of 47.17% and 17.69%, respectively, compared to their corresponding non-supplemented groups (*p* < 0.05). Furthermore, the HFD group exhibited 22.45% and 39.57% higher serum alkaline phosphatase (ALP) activity than the MFD and LFD groups, respectively (*p* < 0.05). Conversely, the MFDV group showed a 23.08% decrease in serum ALP activity relative to the MFD group (*p* < 0.05).

### 3.7. Serum Antioxidant Capacity

Table 2 illustrates the impact of varying dietary lipid levels on the oxidative stress status of *S. fuscescens*. A clear upward trend in serum malondialdehyde (MDA) concentration was observed with increasing dietary fat levels. The low-fat diet (LFD) group exhibited a significantly lower MDA concentration compared to the medium-fat diet (MFD) and high-fat diet (HFD) groups. Additionally, the medium-fat diet with multivitamins (MFDV) and high-fat diet with multivitamins (HFDV) groups demonstrated significantly reduced serum MDA concentrations (*p* < 0.05) relative to the MFD and HFD groups. Serum total antioxidant capacity (T-AOC) showed a decreasing trend, reaching its nadir in the HFD group as dietary lipid concentrations increased. The inclusion of multivitamins consistently enhanced T-AOC activity (*p* < 0.05).

As shown in Table 2, increasing dietary lipid levels led to a decline in serum T-SOD, CAT, and GSH levels. Serum T-SOD activity was significantly elevated (*p* < 0.05) in the MFDV and HFDV groups compared to the LFD, MFD, and HFD groups. Serum CAT activity was significantly higher in the MFDV group (*p* < 0.05) compared to the MFD group, and in the HFDV group (*p* < 0.05) compared with the HFD group. However, no significant differences in oxidized glutathione (GSSG) concentration were observed among the MFD, MFDV, HFD, and HFDV groups.

### 3.8. Serum Immune Parameters

As shown in Table 2, serum MPO activity initially increased and then declined, showing no significant difference between LFD and MFD groups, while the HFD group had significantly lower activity (*p* < 0.05). Serum LZM activity consistently decreased as lipid content increased from 10% to 16% (*p* > 0.05). Opposing trends were observed in serum MPO and LZM activities, with MFDV and HFDV groups showing significantly reduced MPO activity (*p* < 0.05) and increased LZM activity (*p* < 0.05).

Serum C3 concentration significantly increased (*p* < 0.05), and C4 concentration significantly fell (*p* < 0.05) with higher dietary lipid levels. The MFDV group had significantly higher serum C3 concentration compared to the MFD and HFDV groups (*p* < 0.05), while the HFDV group had significantly higher serum C4 concentration than the MFDV and HFD groups (*p* < 0.05). IgM concentration was significantly higher in the LFD group compared to the MFD and HFD groups, and the MFDV and HFDV groups had significantly elevated serum IgM concentration compared to the MFD and HFD groups (*p* < 0.05).

### 3.9. Analysis of Intestinal Histology

As dietary lipid levels increased, there was an observed upward trend in intestinal villus length. The high-fat diet (HFD) group demonstrated significantly greater villus length (*p* < 0.05) compared to the low-fat diet (LFD) and medium-fat diet (MFD) groups. Notably, villus length was significantly reduced in the MFD group (*p* < 0.05) compared to the medium-fat diet with variable lipid levels (MFDV) group. Dietary lipid levels did not significantly impact villus width or crypt depth (refer to Figure S2). The maximum crypt depth was observed in the MFDV group, significantly surpassing that of the other four groups. The thickness of the longitudinal and circular muscle layers initially increased and subsequently decreased with rising dietary lipid content, peaking in the MFD group. In comparison to the MFD group, the MFDV group exhibited a significant increase in the thickness of the longitudinal muscle layer, achieving the highest value among the LFD, MFD, and HFD groups (*p* < 0.05). The number of intestinal goblet cells initially rose and then declined with increasing dietary lipid levels. The MFD group displayed a significantly lower count of intestinal goblet cells compared to the LFD, MFDV, HFD, and high-fat diet with variable lipid levels (HFDV) groups.

## 4. Discussion

In this study, the LFD and MFD groups exhibited lower mean body weight, WGR, SGR, and FER compared to the HFD group. FW in the HFD group was significantly higher than that in the LFD and MFD groups (*p* < 0.05). These findings suggest that increasing dietary lipid levels enhances the growth of *S. fuscescens*, provided that the dietary lipid content does not exceed 16%. Similar findings have been documented in largemouth bass (*Micropterus salmoides*) [22] and golden pompano (*Trachinotus ovatus*) [23]. Furthermore, the HFD group exhibited significantly longer intestinal villus length than the LFD and MFD groups (*p* < 0.05). An enlarged intestinal surface area, which is generally attributed to increased villus length, villus width, and muscle layer thickness, facilitates improved nutrient absorption [24]. The increased intestinal villus length may be associated with enhanced FER, as it enlarges the intestinal surface area and improves nutrient absorption, aligning with observations in *Oncorhynchus mykiss* [25]. This adaptation may contribute to the enhanced growth performance observed in *S. fuscescens* within the HFD group compared to LFD and MFD groups. Moreover, compared to their non-multivitamin counterparts, the MFDV group demonstrated increases in villus length, longitudinal muscle thickness, FER, and overall growth compared to their non-supplemented counterparts. In the HFD group, multivitamin supplementation led to improvements in villus length and longitudinal muscle thickness, although FER remained unchanged. Previous studies have demonstrated that vitamin A deficiency impairs intestinal morphology in ♀*E. fuscoguttatus* × ♂*E. lanceolatus* [26], while dietary vitamin C supplementation enhances villus height and goblet cell numbers in *O. niloticus* [27,28]. Collectively, these findings suggest that multivitamin supplementation may improve intestinal morphology and thereby potentially enhance nutrient absorption and feed utilization in *S. fuscescens*. Furthermore, studies on Caspian trout (*Salmo caspius*) [29], Chinese perch (*Siniperca chuatsi*) [30], and fingerling common carp (*Cyprinus carpio* var. *communis*) [31] have corroborated that vitamin supplementation positively influences growth performance. The growth-promoting effect may be partly associated with improved intestinal morphology, as evidenced by significantly elongated villi that augment the nutrient-absorptive surface area (*p* < 0.05).

In the present study, histopathological examination of *S. fuscescens* revealed a direct correlation between dietary lipid levels and tissue deposition, evidenced by pronounced hepatic vacuolation and substantial lipid droplet accumulation observed in H&E and ORO staining, respectively. This lipid accumulation resulted in increased liver mass, as indicated by elevated HSI, enhanced visceral adiposity, as reflected by increased IPFI, and altered body morphology, evidenced by increased CF, ultimately leading to higher WGR. Similar findings have been reported in Amur sturgeon (*Acipenser schrenckii*) [32] and *M. salmoides* [33] fed with HFD. Notably, multivitamin supplementation ameliorated the histopathological changes, resulting in reduced hepatic vacuolation, lipid droplet accumulation, liver weight, and HSI. Consequently, multivitamins partially alleviated the hepatic metabolic stress induced by HFDs while maintaining relatively higher growth performance in *S. fuscescens*. Similar outcomes have been documented in *M. salmoides*, black sea bream (*Acanthopagrus schlegeli*), and ♀*E. fuscoguttatus* × ♂*E. lanceolatus* [34,35,36].

The defensive mechanisms against oxidative stress are primarily dependent on SOD, CAT, and GSH-Px. SOD catalyzes the conversion of superoxide anions into hydrogen peroxide (H_2_O_2_) [37]. Subsequently, CAT and GSH-Px work synergistically to neutralize H_2_O_2_; CAT catalyzes its decomposition into water (H_2_O) and oxygen (O_2_), while GSH-Px utilizes glutathione (GSH) to eliminate reactive oxygen species (ROS) and inhibit lipid peroxidation [38]. Lipid peroxidation results in the formation of MDA, a byproduct whose increased levels are indicative of cellular membrane damage. T-AOC provides a holistic evaluation of both enzymatic and non-enzymatic antioxidant systems [39]. In the present study, darkened *S. fuscescens* demonstrated marked reductions in serum T-SOD and T-AOC levels, alongside significant elevations in oxidized glutathione (GSSG) and MDA concentrations (Figure 2 and Figure S1). These findings collectively suggest impaired antioxidant defenses and systemic oxidative stress compared to healthy controls. Kaveh [40] observed a decline in serum antioxidant capacity in *O. mykiss* (Walbaum, 1792) infected with *Ichthyophthirius*, which was accompanied by increased activities of ALT, AST, and ALP, indicating of hepatic dysfunction. Similar findings have been reported in hybrid sturgeon (♀*Acipenser baerii* × ♂*A. schrenckii*) infected with *Staphylococcus saprophyticus* [41] and in *O. mykiss* infected with viral hemorrhagic septicemia [42]. These studies indicate that the prevalence of darkening syndrome may rise in association with liver damage and reduced antioxidant capacity, aligning with the increased incidence of darkening syndrome observed in *S. fuscescens* fed with HFDs. In this study, elevated dietary lipid levels were correlated with significant increases in serum MDA, AST, and ALT levels, accompanied by concurrent declines in T-SOD, CAT, and GSH levels. At comparable dietary lipid concentrations, multivitamin supplementation was found to significantly decrease serum levels of MDA, AST, and ALT, while substantially increasing T-AOC, T-SOD, CAT, and GSH levels (*p* < 0.05). This indicates an enhancement in antioxidant capacity and a reduction in liver damage in *S. fuscescens*. Similar effects have been documented in turbot (*Scophthalmus maximus*) [43] and Chinese mitten crab (*Eriocheir Sinensis*) [44] when fed HFDs supplemented with vitamins A and E. These collective findings, alongside the reduced incidence of darkening syndrome in *S. fuscescens* fed HFDV and MFDV compared to those on HFD and MFD alone, suggest that dietary multivitamin supplementation may decrease the occurrence of darkening syndrome by enhancing antioxidant capacity and alleviating hepatic damage.

The innate immunological capacity of fish is evidenced by serum complement levels and the activities of MPO and LZM. The complement system is a vital component of vertebrate innate immunity, consisting of plasma proteins and their receptors [45]. The classical pathway enhances humoral immunity by facilitating opsonophagocytosis and forming the membrane attack complex through the proteolytic products of C3 and C4 [46]. HFDs elevate C3 levels, with C3 production showing a positive correlation with adipose tissue mass [47,48]. Additionally, C3 and other complement components act as key initiators of obesity-associated low-grade inflammation [47]. MPO, released during the respiratory burst, utilizes H_2_O_2_ to oxidize substrates, thereby inflicting damage on pathogenic microorganisms [49]. LZM is an immunological molecule that activates the complement system and is crucial for innate immunity [50]. As a key component of specific humoral immunity in teleosts, IgM functions as a dependable biomarker for evaluation [51]. In this study, a significant reduction in serum levels of MPO, LZM, and C4, alongside a notable elevation in serum IgM levels, collectively suggests impaired immune function and the presence of infection in darkened *S. fuscescens* (Figure S1). The observed anorexic behavior and intestinal morphological damage may be linked to these changes in immune-related serum parameters, aligning with the findings of Duan et al. [52], who documented that decreased feeding rates in Koi (*C. carpio* var. *koi*) compromise antioxidant and immune capacities. With an increase in dietary lipid content, serum C3 levels in *S. fuscescens* exhibited an upward trend, whereas LZM levels declined. The HFD group displayed significantly lower serum C4 levels and MPO activity compared to LFD and MFD groups. Notably, serum LZM levels were significantly higher in MFDV and HFDV groups compared with the MFD and HFD groups. HFDs have been reported to suppress immune function in various fish species, including *I. punctatus* [53] and *M. salmoides* [51]. Conversely, the immunoenhancing effects of vitamin-fortified diets have been observed in species such as sub-adult turbot (*S. maximus*) [54]. Collectively, these findings across multiple fish species suggest that lipid-induced immunosuppression is a prevalent metabolic risk in aquaculture, for which dietary vitamin supplementation serves as an effective strategy to maintain the health of aquaculture fish.

Under the conditions of this study, we found that as dietary lipid content increased, the risk of darkening syndrome in *S. fuscescens* increased and survival decreased. However, even at relatively high dietary lipid levels, the multivitamins-supplemented group still showed excellent mitigating effects. Notably, the deteriorating trends in hepatic histological and serological indices caused by high-lipid diets were largely opposite to the improving trends in these indices after multivitamins supplementation. Combined with the serological differences between healthy and diseased fish and the antioxidant properties of the supplemented vitamins, these results seem to suggest a hypothesis: high-lipid-induced oxidative stress exacerbates the occurrence of darkening syndrome in *S. fuscescens*. Although high-lipid diets are rarely used in actual aquaculture production, this study suggests that the rationality of current dietary lipid levels should be considered in *S. fuscescens* culture. High lipid may merely be a source of oxidative stress stimulation rather than a direct cause of the syndrome. It is recommended to further conduct multi-gradient experiments on fat levels and fishmeal replacement levels, comprehensively considering factors such as feed cost, the incidence of darkening syndrome, and yield, so as to screen for the most cost-effective feed formula, and future studies should also verify the preventive effects of multivitamins or other antioxidants under other rearing conditions and elucidate the specific mechanisms of oxidative stress in this syndrome through molecular biology research.

## 5. Conclusions

An HFD was found to enhance growth performance in *S. fuscescens*; however, it also induced adverse metabolic effects. These detrimental changes were associated with alterations in serum parameters characteristic of darkening syndrome, suggesting that HFD contributed to the onset of this condition. In contrast, multivitamin supplementation was linked to a reduced incidence of darkening syndrome and an amelioration of the negative effects associated with a high-lipid diet. Therefore, when the darkening syndrome occurs under production conditions, it may be worthwhile to consider lowering the dietary lipid level and supplementing antioxidant-containing multivitamins as a potential mitigation strategy.

## Figures and Tables

**Table 1:** Growth performance and morphological indices in different dietary groups of *S. fuscescens*.

Parameters	LFD	MFD	MFDV	HFD	HFDV
IW (g)	70.06 ± 1.69 a	68.89 ± 1.66 a	69.76 ± 1.69 a	69.78 ± 1.44 a	69.95 ± 1.34 a
FW (g)	77.82 ± 1.88 a	80.41 ± 1.69 ab	85.22 ± 1.72 b	92.25 ± 1.68 c	86.19 ± 1.41 bc
WGR (%)	14.19 ± 3.49 a	21.38 ± 4.28 ab	27.22 ± 4.62 ab	35.84 ± 3.94 b	25.76 ± 3.10 ab
SGR (%/day)	0.19 ± 0.05 a	0.28 ± 0.06 a	0.37 ± 0.06 ab	0.50 ± 0.05 b	0.38 ± 0.04 ab
FER (g/g)	1.73 ± 0.19 a	2.14 ± 0.17 ab	3.09 ± 0.40 bc	3.80 ± 0.04 c	2.80 ± 0.15 abc
Food intake (g/day)	1589 ± 58.28 a	1914 ± 71.86 bc	1784 ± 64.70 ab	2097 ± 87.11 c	2060 ± 91.67 bc
Feed rate (%)	11.06 ± 0.38 a	12.79 ± 0.42 b	11.95 ± 0.37 a	12.92 ± 0.44 b	13.32 ± 0.52 b
Liver weight (g)	1.94 ± 0.11 ab	1.95 ± 0.10 ab	1.88 ± 0.08 b	2.25 ± 0.11 a	2.02 ± 0.07 ab
HSI (%)	2.32 ± 0.04 a	2.34 ± 0.03 a	2.32 ± 0.02 a	2.40 ± 0.02 a	2.37 ± 0.02 a
VSI (%)	11.61 ± 0.09 a	11.57 ± 0.07 ab	11.60 ± 0.06 ab	11.34 ± 0.05 b	11.48 ± 0.05 ab
CF (g/cm^3^)	1.33 ± 0.02 a	1.33 ± 0.02 a	1.33 ± 0.01 a	1.38 ± 0.01 a	1.35 ± 0.01 a
IPFI (%)	0.83 ± 0.12 ab	0.75 ± 0.13 ab	0.68 ± 0.11 b	1.17 ± 0.11 a	0.86 ± 0.11 ab
ISI (%)	7.25 ± 0.23 a	7.31 ± 0.17 a	7.23 ± 0.14 a	7.85 ± 0.12 a	7.49 ± 0.12 a

Note: Values are presented as mean ± SEM (*n* = 3). Statistical analysis was performed using One-way ANOVA followed by Tukey’s multiple range test, with significance level set at α = 0.05. Values sharing identical lowercase letters indicate no statistically significant differences (*p* > 0.05) between groups, whereas different letters represent significant differences (*p* < 0.05). Abbreviations: IW, initial weight; FW, final weight; WGR, weight gain rate; SGR, specific growth rate; FER, feed efficiency rate; HSI, hepatosomatic index; VSI, viscerosomatic index; CF, condition factor; IPFI, intraperitoneal fat index; ISI, intestosomatic index.

**Table 2:** Serum parameters in different dietary groups of *S. fuscescens*.

Parameters	LFD	MFD	MFDV	HFD	HFDV
ALT (U/L)	2115.20 ± 27.86 a	2352.00 ± 12.53 b	1226.00 ± 12.57 c	2417.20 ± 23.64 b	1479.20 ± 6.93 d
AST (U/L)	117.00 ± 4.16 a	232.20 ± 2.54 b	122.80 ± 7.14 a	382.40 ± 4.99 c	314.00 ± 1.82 d
ALP (U/L)	84.40 ± 0.81 a	96.20 ± 4.69 a	74.00 ± 4.17 b	117.80 ± 0.92 c	119.00 ± 4.84 c
MDA (nmol/mL)	25.73 ± 0.42 a	48.16 ± 0.33 b	28.37 ± 0.66 c	88.27 ± 0.76 d	57.04 ± 0.67 e
T-AOC (U/mL)	26.49 ± 0.64 a	18.25 ± 1.34 b	35.47 ± 0.63 c	15.17 ± 1.39 b	27.13 ± 0.65 a
CAT (U/mL)	26.59 ± 0.73 a	17.56 ± 0.59 b	28.71 ± 0.26 a	14.65 ± 0.42 c	19.28 ± 0.63 b
T-SOD (U/mL)	35.47 ± 2.53 a	28.66 ± 1.44 b	59.38 ± 0.96 c	24.26 ± 0.89 b	44.40 ± 1.35 d
GSH-Px (U/mL)	516.12 ± 20.38 a	434.97 ± 27.63 b	697.24 ± 3.07 c	462.19 ± 2.76 ab	404.29 ± 19.29 b
GSH (mg/L)	255.06 ± 3.33 a	188.84 ± 5.05 b	249.23 ± 10.61 a	136.21 ± 2.61 c	171.23 ± 5.34 b
GSSG (μmol/L)	2.28 ± 0.29 a	3.04 ± 0.11 ab	2.90 ± 0.19 ab	3.79 ± 0.46 b	2.81 ± 0.27 ab
C3 (μg/L)	4.44 ± 0.26 a	7.12 ± 0.37 b	9.92 ± 0.51 c	8.43 ± 0.30 bc	4.13 ± 0.33 a
C4 (μg/L)	28.52 ± 0.34 a	24.27 ± 0.88 bd	22.66 ± 0.74 b	19.72 ± 0.56 c	25.55 ± 0.46 d
IgM (μg/L)	2.41 ± 0.06 a	1.96 ± 0.04 b	2.73 ± 0.03 c	2.04 ± 0.02 b	2.32 ± 0.07 a
MPO (U/L)	114.45 ± 5.00 a	122.71 ± 6.23 a	50.93 ± 2.42 b	83.19 ± 3.58 c	47.98 ± 1.69 b
LZM (U/mL)	82.05 ± 2.81 a	70.77 ± 3.40 a	108.72 ± 3.77 b	62.56 ± 4.41 a	107.69 ± 9.86 b

Note: Values are presented as mean ± SEM (*n* = 5 per group). Statistical analysis was performed using One-way ANOVA followed by Tukey’s multiple range test, with significance level set at α = 0.05. Values sharing identical lowercase letters indicate no statistically significant differences (*p* > 0.05) between groups, whereas different letters represent significant differences (*p* < 0.05). Abbreviations: ALT, alanine aminotransferase activity; AST, aspartate aminotransferase activity; ALP, alkaline phosphatase activity; C3, complement 3 concentration; C4, complement 4 concentration; IgM, immunoglobulin M concentration; CAT, catalase activity; T-SOD, total superoxide dismutase activity; T-AOC, total antioxidant capacity activity; GSH-Px, glutathione peroxidase activity; MDA, malondialdehyde concentration; GSSG, oxidized glutathione concentration; GSH, reduced glutathione concentration; MPO, myeloperoxidase activity; LZM, lysozyme activity.

## Data Availability

Data are contained within the article or Supplementary Materials. Further inquiries can be directed to the corresponding authors.

## Supplementary Materials

The following supporting information can be downloaded at https://www.mdpi.com/article/10.3390/ani16213495/s1, Figure S1: Other serum biochemical parameters of normal *S. fuscescens* and those affected by darkening syndrome; Figure S2: Intestinal histology and morphological parameters of *S. fuscescens*; Table S1: Composition of experimental diets (% dry matter).
