# Supplementary material for: Dietary Multivitamin Supplementation in Siganus fuscescens: Integrated Effects on Growth Performance, Antioxidant Capacity, and Mitigation of Darkening Syndrome Under High-Lipid Dietary Stress
Source: Animals (Basel). 2026 Nov 5;16(21):3495. doi: 10.3390/ani16213495 (PMC13762004; doi:10.3390/ani16213495)
Supplement: Supplementary file 1 [file animals-16-03495-s001.zip › animals-4640918-supplementary.pdf]

Table S1. Composition of experimental diets (% dry matter).

Items	Groups				
	LFD	MFD	MFDV	HFD	HFDV
Ingredients					
Soy protein concentrate†	22	22	22	22	22
Fish meal†	20.4	20.4	20.4	20.4	20.4
Shrimp meal†	8.8	8.8	8.8	8.8	8.8
Soybean meal†	6.8	6.8	6.8	6.8	6.8
Extruded soybean meal†	5.2	5.2	5.2	5.2	5.2
Fermented soybean meal†	3.2	3.2	3.2	3.2	3.2
Soybean oil†	4.8	6.8	6.8	8.8	8.8
Standardized phospholipids†	2.2	2.2	2.2	2.2	2.2
Fish oil†	2	3	3	4	4
Tapioca starch†	14	11	10	8	7
Wheat flour†	3.6	3.6	3.6	3.6	3.6
Carrageenan†	0.6	0.6	0.6	0.6	0.6
Multivitamin‡	0	0	1	0	1
Premix§	6.4	6.4	6.4	6.4	6.4
Crude protein	40.76	40.60	40.11	40.34	40.44
Crude lipid	10.39	13.46	13.33	16.43	16.49
Crude ash	7.92	7.88	7.79	7.81	7.91
Moisture	7.86	7.91	8.02	7.90	7.95

† Soy protein concentrate, fish meal, shrimp meal, soybean meal, extruded soybean meal, fermented soybean meal, soybean oil, standardized phospholipids, fish oil, tapioca starch, wheat flour, and carrageenan were obtained from Fuzhou Haima Feed Co., Ltd. (Fuzhou, China).

‡ Multivitamin supplement primarily contained vitamins A, C, and E.

§ Premix composition per kg of diet: Vitamin B1, 16 mg; Vitamin B2, 27 mg; Vitamin B6, 10 mg; Vitamin B12, 0.08 mg; Vitamin K3, 10 mg; Inositol, 1100 mg; Nicotinamide, 78 mg; Folic acid, 4 mg; Vitamin E, 90 mg; Vitamin A, 5,000 IU; Vitamin D3, 1,600 IU; Vitamin C, 140 mg; Mg, 127.8 mg; Mn, 4.34 mg; Zn, 6.429 mg; Cu, 1.5 mg; Fe, 25 mg; Se, 0.05 mg; Co, 0.09 mg; I, 0.5 mg.

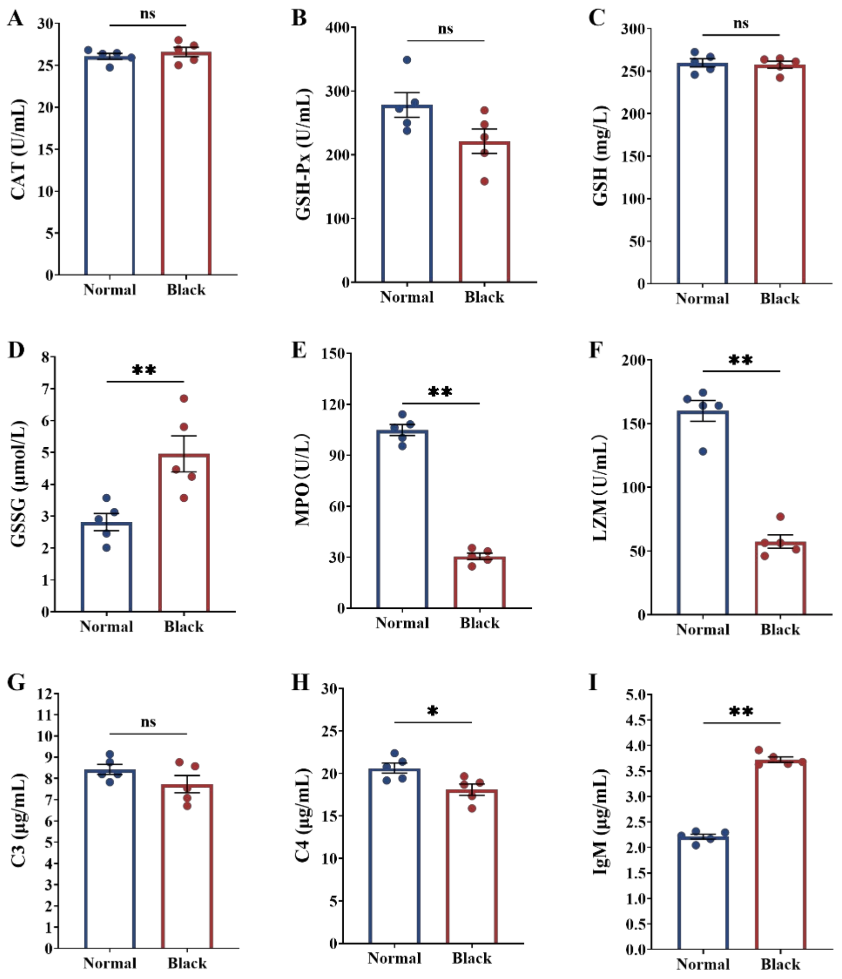

Figure S1 Other serum biochemical parameters of normal *S. fuscescens* and those affected by darkening syndrome; (A) CAT, catalase activity; (B) GSH-Px, glutathione peroxidase activity; (C) GSH, reduced glutathione concentration; (D) GSSG, oxidized glutathione concentration; (E) MPO, myeloperoxidase activity; (F) LZM, lysozyme activity; (G) C3, complement 3 concentration; (H) C4, complement 4 concentration; (I) IgM, immunoglobulin M concentration; Data are expressed as mean ± SEM (n=5); Statistical analysis was performed using Student’s t-test, with significance level set at α=0.05; ns, not significant; *$p < 0.05$; **$p < 0.01$.

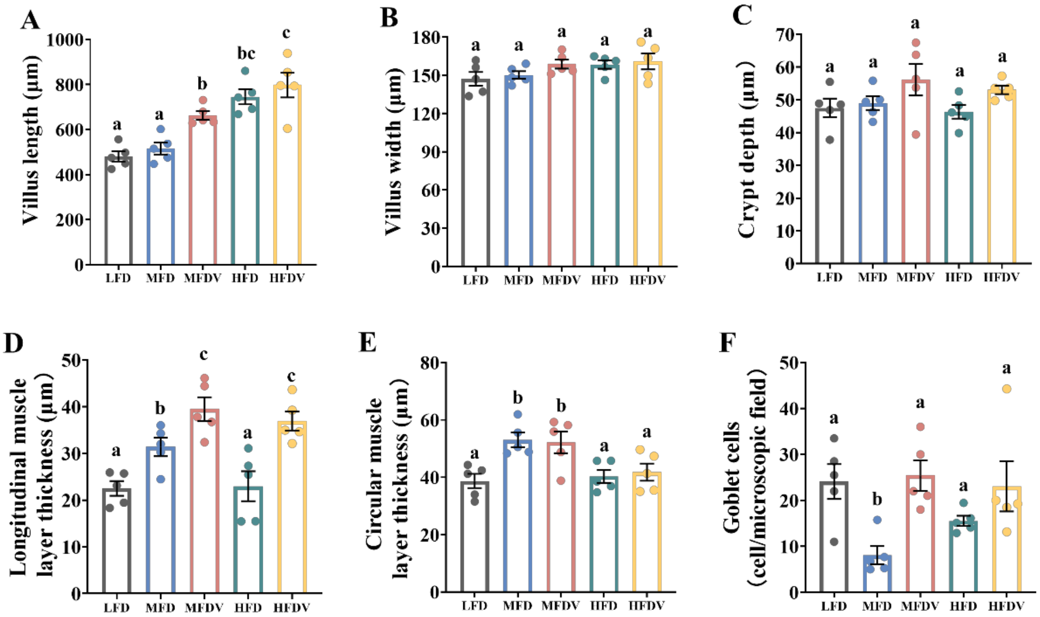

Figure S2. Intestinal histology and morphological parameters of *S. fuscescens*: (A) Villus length; (B) Villus width; (C) Crypt depth; (D) Thickness of longitudinal muscle layer; (E) Thickness of circular muscle layer; (F) Number of goblet cells in intestinal villi. Data are expressed as mean ± SEM (n=5 per group); Statistical analysis was performed using One-way ANOVA followed by Tukey's multiple range test, with significance level set at α=0.05. Values sharing identical lowercase letters indicate statistically non-significant differences ($p > 0.05$), whereas different letters represent significant differences ($p < 0.05$).
